# Adenoviral vectors for cardiovascular gene therapy applications: a clinical and industry perspective

**DOI:** 10.1007/s00109-022-02208-0

**Published:** 2022-05-24

**Authors:** Schwartze JT, Havenga M, Bakker WAM, Bradshaw AC, Nicklin SA

**Affiliations:** 1 Institute of Cardiovascular and Medical Sciences, University of Glasgow, Glasgow, UK; 2 Batavia Biosciences B.V., Bioscience Park Leiden, Zernikedreef 16, 2333 CL Leiden, The Netherlands

**Keywords:** Adenovirus, Gene therapy, Cardiovascular disease, Industry, Good manufacturing practices

## Abstract

**Abstract:**

Despite the development of novel pharmacological treatments, cardiovascular disease morbidity and mortality remain high indicating an unmet clinical need. Viral gene therapy enables targeted delivery of therapeutic transgenes and represents an attractive platform for tackling acquired and inherited cardiovascular diseases in the future. Current cardiovascular gene therapy trials in humans mainly focus on improving cardiac angiogenesis and function. Encouragingly, local delivery of therapeutic transgenes utilising first-generation human adenovirus serotype (HAd)-5 is safe in the short term and has shown some efficacy in drug refractory angina pectoris and heart failure with reduced ejection fraction. Despite this success, systemic delivery of therapeutic HAd-5 vectors targeting cardiovascular tissues and internal organs is limited by negligible gene transfer to target cells, elimination by the immune system, liver sequestration, off-target effects, and episomal degradation. To circumvent these barriers, cardiovascular gene therapy research has focused on determining the safety and efficacy of rare alternative serotypes and/or genetically engineered adenoviral capsid protein-modified vectors following local or systemic delivery. Pre-clinical studies have identified several vectors including HAd-11, HAd-35, and HAd-20–42-42 as promising platforms for local and systemic targeting of vascular endothelial and smooth muscle cells. In the past, clinical gene therapy trials were often restricted by limited scale-up capabilities of gene therapy medicinal products (GTMPs) and lack of regulatory guidance. However, significant improvement of industrial GTMP scale-up and purification, development of novel producer cell lines, and issuing of GTMP regulatory guidance by national regulatory health agencies have addressed many of these challenges, creating a more robust framework for future adenoviral-based cardiovascular gene therapy. In addition, this has enabled the mass roll out of adenovirus vector-based COVID-19 vaccines.

**Key messages:**

- First-generation HAd-5 vectors are widely used in cardiovascular gene therapy.
- HAd-5-based gene therapy was shown to lead to cardiac angiogenesis and improved function.
- Novel HAd vectors may represent promising transgene carriers for systemic delivery.
- Novel methods allow industrial scale-up of rare/genetically altered Ad serotypes.
- National regulatory health agencies have issued guidance on GMP for GTMPs.

## Introduction

The concept of gene therapy was first proposed many decades ago and pre-dates the completion of the Human Genome Project. In theory, the concept is simple: once a disease-causing mutation in the genetic code is identified, delivery of a correct copy would provide a cure. This paradigm has rapidly gained traction for inherited, monogenic disorders, which are caused by mutations in a single gene and are estimated to affect 1 in 100 people worldwide [1]. In addition, investigation of underlying pathomolecular mechanisms of cell and organ function has identified specific candidate genes which become deregulated in disease [2, 3]. For example, restoration of target gene expression via gene therapy is being explored to tackle heart failure (HF) [2, 3]. However, the gene therapy community has been faced with substantial challenges in obtaining sufficient levels of protein expression from therapeutic genes in target organs, using safe and effective gene delivery vectors. Therefore, many decades of work have focussed on identifying, researching, and refining a range of both viral and non-viral gene therapy vectors to ensure they are safe and efficient.

One of the earliest identified viruses explored as a gene delivery vector is human adenovirus serotype 5 (HAd-5), and since then, this serotype has been widely studied for its ability to deliver therapeutic genes for treatment of inherited [4–6] and acquired diseases [7–12]. In cardiovascular disease, adenoviral-mediated gene therapy has been investigated in experimental models of atherosclerosis [7], myocardial ischaemia [8], restenosis and vein graft failure (VGF) [9], pulmonary arterial hypertension (PAH) [4], and essential hypertension [10]. As one of the earliest developed vectors, HAd-5 has been assessed in many clinical trials, including cardiovascular applications in myocardial ischaemia, therapeutic angiogenesis, and HF [2, 13–17]. Here, another hurdle in translating experimental gene therapy procedures to the clinic has been the development of robust, good manufacturing practice (GMP), and release processes that comply with regulatory requirements. Collaboration between academia and industry has played a key role in addressing and overcoming these challenges. Owing to the combined efforts of public and private sector partners, the adenoviral vector platform has matured tremendously such that multiple adenoviral vector-based vaccines are currently at the forefront of the fight against COVID-19, caused by severe acute respiratory syndrome coronavirus-2 (SARS-CoV-2) [18–24]. Here, the development of adenoviral vectors for cardiovascular disease and vaccine applications is reviewed, and we provide an industry perspective on the achievements and challenges in manufacturing and scale-up, as well as discussing clinical application of these versatile gene delivery vectors.

### Adenovirus structure and genome organisation

**Fig. 1:**
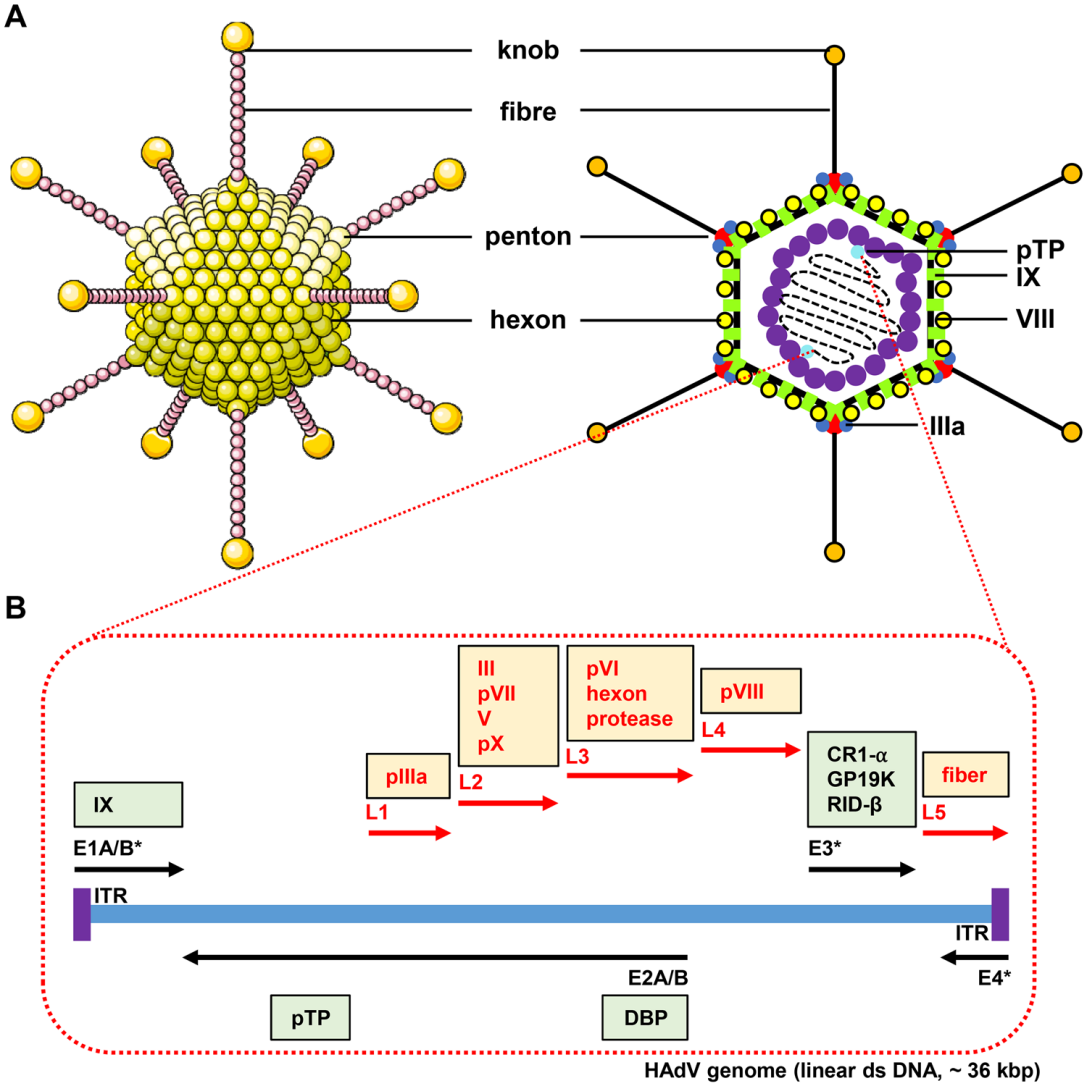
Ad structure and genome organisation. (**A**) Adenoviral virions are non-enveloped icosahedral-shaped capsids ranging from 70 to 90 nm in diameter [202]. Each capsid encompasses a total of 252 proteins classified into 240 trimeric hexons, 12 pentameric penton bases, and 12 trimeric fibre proteins (reviewed in) [203]. (**B**) The capsid contains linear double-stranded (ds) DNA ranging from 26 to 46 kb. The Ad genome is divided into 4 early (E) and 5 late (L) transcriptional units. Early transcriptional units encode non-structural proteins which regulate Ad DNA replication and host cell metabolism [58]. Late transcriptional units encode structural proteins which form the Ad virion. *indicates regions which are often manipulated/deleted to generate HAd gene therapy vectors. Abbreviations: IX, gene-encoding capsid protein IX; pIIIa, gene-encoding capsid protein precursor pIIIa; III, gene-encoding penton base; pVII, gene-encoding core protein precursor VII; V, gene-encoding core protein V; pVI, gene-encoding capsid protein precursor VI; pVIII, gene-encoding capsid protein precursor VIII; CR1-α, gene-encoding membrane glycoprotein E3 CR1-α; GP19K, gene-encoding membrane glycoprotein E3 gp19K; RID-β, membrane protein E3 RID-β; ITR, inverted terminal repeat; pTP, gene-encoding pre-terminal protein; DBP, gene-encoding DNA-binding protein

Adenoviruses are classified under the family of Adenoviridae which is subdivided into 5 distinct generations: *Mastadenovirus*, *Aviadenovirus*, *Siadenovirus*, *Atadenovirus*, and *Ichtadenovirus* (reviewed in) [25]. Human adenoviruses belong to the generation of mastadenoviridae which are subdivided into 7 subgroups (A–G) with a total number of 67 known serotypes (reviewed in) [26]. A schematic representation of Ad structure and genome organisation is presented in Fig. 1.

In humans, wild-type HAds typically cause conjunctivitis, keratoconjunctivitis, upper and lower respiratory tract infections, pneumonia, gastroenteritis, hepatitis, and cystitis [27]. Adenoviruses infect a broad range of species and include simian Ads, bovine Ads, porcine Ads, ovine Ads, canine Ads, murine Ads, and fowl Ads [28].

## The evolution of different Ad vector systems

The in-depth understanding of Ad biology and genome organisation allowed for the engineering of the Ad genome, thus enabling the development of a large variety of human and non-human Ad vectors capable of delivering transgenes to target cells and tissues. Based on specific viral gene deletion, Ad vectors are typically classified as first-, second-, or third-generation vectors. In addition, conditionally replicating or oncolytic Ads have been designed, to selectively replicate in, and destroy tumour cells. A summary of the genetic engineering underlying these different vectors is provided below.

First-generation Ad vectors are non-enveloped icosahedral-shaped capsids which are made up of trimeric hexons, pentameric penton bases, and trimeric fibre proteins (Fig. 1A). Together, these structures protect the double-stranded adenoviral DNA and facilitate infection of target host cells. In first-generation Ad vectors, the E1 and often E3 regions are substituted for an expression cassette with an insert capacity of 8.2 kb (reviewed in) [29, 30] (Fig. 1B). Genes within the E1 region encode proteins which orchestrate viral replication and promote host cell proliferation (reviewed in) [30–32]. The development of the HEK293 cell line, which contains Ad DNA encoding the E1 region, provided a crucial helper cell line able to provide key replicative functions of the virus in *trans* to enable laboratory amplification and production of replication-deficient vectors [33]. In contrast, the E3 region contains genes encoding proteins that modulate the immune response following wild-type Ad infection [34]. These functions are only activated when E1 is functional, and, hence, E3 is dispensable when designing an Ad as a gene therapy vector.

Owing to their intrinsic ability to trigger strong cellular and humoral immune responses, Ad-based vectors are widely studied as vaccine vectors. Non-replicating human and non-human Ad-based vaccine vectors include Ad26.COV2.S [22], simian ChAdOx1 nCoV-19 [18, 21, 24], Gam-COVID-Vac (Sputnik V, recombinant Ad26 and Ad5) [20, 23], Ad26.ZEBOV/MVA-BN®-Filo [35], and Ad26.Mos4.HIV [36] (Table 2). In addition, an Ad-5-based vector encoding the spike “S” and nucleoside “N” genes of SARS-CoV-2 has been reported to elicit a mucosal immune response in the digestive tract when given orally as a tablet [37].

Second-generation Ad vectors feature a combination of E1/E3 deletions with E2 and/or E4 deletions and have proven less immunogenic compared to first-generation vectors (reviewed in) [38]. The E2 region encodes for the DNA-binding protein (DBP), the pre-terminal protein (pTP), and the viral DNA polymerase which are crucial for viral replication (reviewed in) [39]. The development of this generation of adenoviral gene therapy vector has been hampered by the lack of efficient helper cell lines co-expressing E1 and E2 functions. The E4 region contains genes which modulate cell signalling, the cell cycle, and DNA repair (reviewed in) [40]. Following deletion of the E1, E2, E3, and E4 regions, second-generation vectors have a maximal insert capacity of 14 kb (reviewed in) [30]. The scientific and industrial interest in second-generation vectors have waned as they have largely been surpassed using third-generation adenoviral vectors.

Third-generation or helper-dependent (HD) Ad vectors lack all viral genes except two inverted terminal repeats (ITRs) and the packaging signal, thereby allowing a maximal insert size of 36 kb [41, 42]. HD-Ad propagation requires an additional E1-deleted helper virus (HV) which provides all viral proteins crucial for the rescue of the HD Ad. Third-generation Ad vectors are sophisticated gene delivery systems capable of mediating long-term gene delivery and therapeutic benefit; however, they are challenging to translate to GMP-grade manufacturing due to the requirement to ensure that HVs do not contaminate the final vector batches.

Conditionally replicating or oncolytic Ads have the ability to directly target and destroy cancer cells due to the lytic nature of replicating Ads (reviewed in) [38]. Intact E1A and E1B regions are crucial for Ad replication in healthy host cells which enable the virus to block host cell defence mechanisms mediated through P53 and retinoblastoma (rb) signalling. In contrast, these regions are dispensable in tumour cells with defective p53 and/or rb. Hence, manipulation/deletion of specific E1A or E1B genes enables targeted Ad replication in cancer cells and subsequent lytic destruction [43, 44]. For example, deletion of the E1B 55 kDa gene prevents viral inactivation of p53 [45], while deletion of 24 bp in the E1A gene leads to translation of a mutant E1A protein which is unable to bind and inhibit rb [46]. Targeted tumour cell lysis may also be achieved by tissue-specific, promoter-driven transcriptional control of the E1 region [47, 48]. Oncolytic HAd-5-based GTMPs approved by the China Food and Drug Administration include Oncorine (rAd5-H101) and Gendicine (rAd-p53) [49, 50] (Table 2).

## HAd-5-dependent transgene delivery

**Fig. 2:**
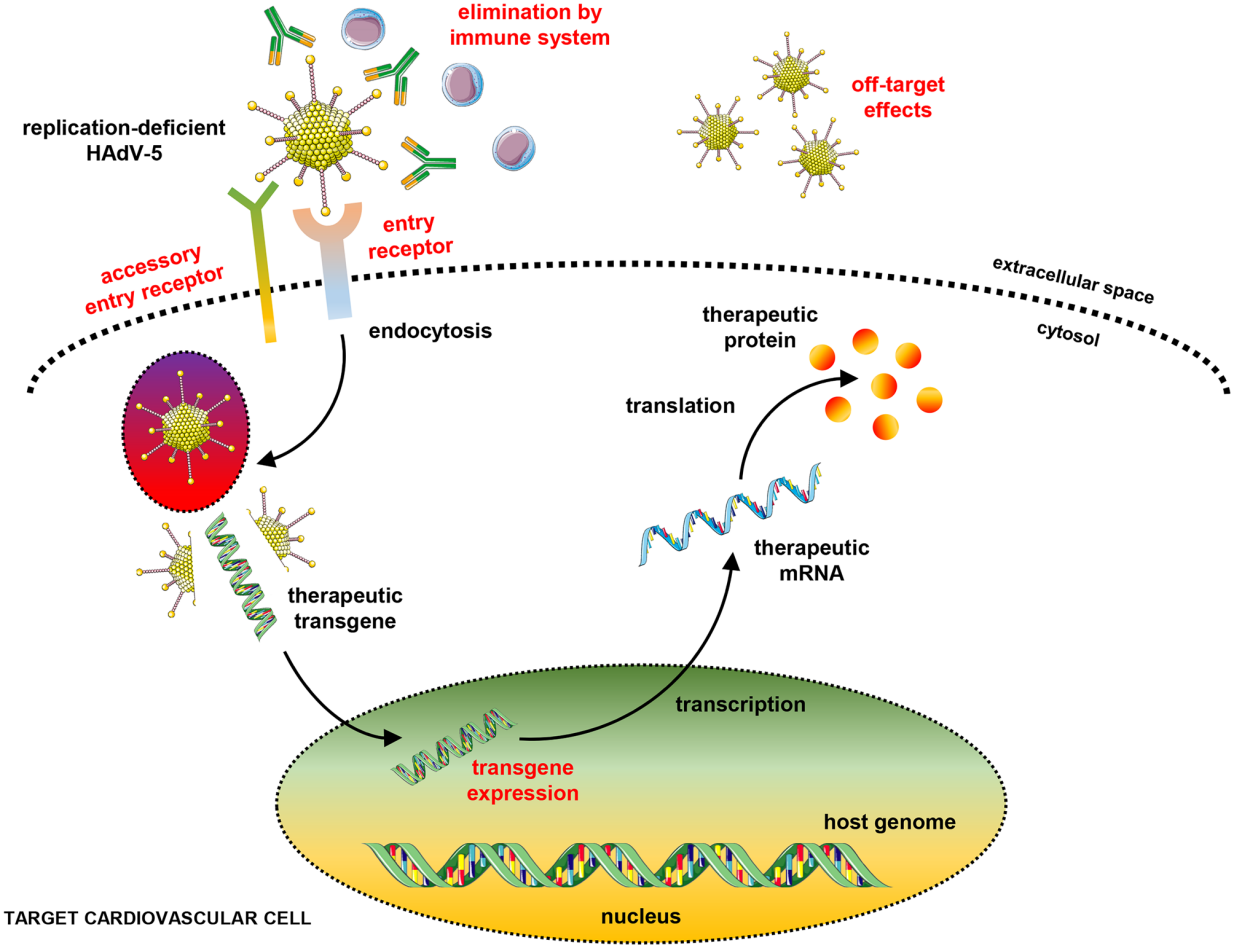
Schematic representation of HAd-5-dependent transgene delivery. Replication-deficient HAd-5 enters a target cardiovascular cell via an entry ± accessory entry receptor. Following receptor-mediated endocytosis, the HAd-5 capsid is broken down, and the viral DNA is imported into the nucleus via nuclear core complexes. The transgene remains extrachromosomal and produces a therapeutic protein. Clinical limitations following HAd-5 delivery include viral elimination by the immune system, off-target effects and hepatotoxicity, reduced transduction efficiency based on tissue-dependent entry/accessory entry receptor density, and transient/loss of transgene expression because of episomal degradation

HAd-5 belongs to HAd subgroup C [51] and represents the preferred vector in many cardiovascular gene therapy trials (Table 1). HAd-5 utilises a range of cell surface receptors for attachment and internalisation, including the coxsackie and adenovirus receptor (CAR) [52], heparan sulphate proteoglycans (HSPG) [53], major histocompatibility complex (MHC)-I [54], vascular cell adhesion molecule (VCAM)-1 [55], and integrins [56] (Fig. 2). Following receptor-mediated endocytosis [56], the viral capsid gradually disassembles allowing binding to the nuclear pore complex and viral DNA import into the nucleus [57]. Nuclear uptake of viral DNA initiates transcription of early units followed by transcription of late units (reviewed in) [58]. In wild-type Ads, replicated viral genomes are packaged into fully functional viral capsids, culminating in virus-induced cell lysis followed by viral release [38]. E1 deletions in the HAd-5 prevents replication in healthy wild-type host cells. E3 is not required for the function of the replication-deficient HAd-5 vector, and its deletion creates additional space for incorporation of larger expression cassettes. The E1 and/or E3 regions are often substituted for an expression cassette which contains a transgene of interest. Following delivery to a target cell the transgene is expressed in the nucleus, remains extrachromosomal, and produces a therapeutic protein [59].

**Table 1:** Advantages and disadvantages of different viral vector systems for cardiovascular gene therapy

Features	1st-generation HAd-5	Adeno-associated virus	Lentivirus
Genome /size	dsDNA, ~ 36 kb	ssDNA, ~ 4.7 kb	ssRNA, ~ 9 kb
Packaging capacity insert	8.2 kb	4.6 kb	8 kb
Infection	Most dividing and non-dividing cells	Most dividing and non-dividing cells	Most dividing and non-dividing cells
Transgene expression	Transient	Transient and/or stable expression	Stable expression
Risk of mutagenesis	-Low to none -Viral DNA / transgene remains episomal	-Yes -Viral DNA / transgene remains episomal	-Yes -Viral genome integrates into host DNA
Immunogenicity	High	Moderate	Low
Neutralising antibodies	Common, high prevalence	Common, high prevalence	Rare, low to no prevalence
Up-scaling/infectious titre	Well established following GMP	Challenging	Challenging 1.97 × 10^9^ transducing units [198]
Advantages relative to comparative viral vectors	-Low to no risk of mutagenesis -Industrial GMP-grade up-scaling well established	-Enables stable long-term transgene expression -Preferred vector for myocardial gene transfer (serotypes 1, 6, 8, and 9) -AAVs have never been shown to cause human disease -Low mutagenesis risk	-Enables stable long-term transgene expression -Reduction in immune-mediated elimination 2^nd^ to low immunogenicity and absence of nAbs
Disadvantages relative to comparative viral vectors	-Transient transgene expression -High immunogenicity and high prevalence of nAbs drive rapid immune-mediated elimination -Hepatic sequestration following intravenous administration	-Low packaging capacity -High prevalence of nAbs risks rapid immune-mediated elimination -Challenging GMP-grade up-scaling	-Heightened risk of mutagenesis -Challenging GMP-grade up-scaling

*AAV* adeno-associated virus, *GMP* good manufacturing practice, *ds* double stranded, *ss* single stranded, *LV* lentivirus vector, *nAbs* neutralising antibodies

## HAd-5-based vectors: advantages and clinical challenges for gene therapy

The continued use of HAd-5-based gene therapy vectors for in vitro and in vivo applications is based on several advantages. The HAd-5 genome has been extensively characterised, which has facilitated genetic engineering and enabled reproducible HAd-5 genome manipulation and/or therapeutic transgene insertion [60]. HAd-5 engages intracellular trafficking pathways that can rapidly deliver therapeutic transgenes to the nucleus, facilitating efficient transgene expression. Furthermore, HAd-5 demonstrates wide tropism for quiescent and non-quiescent cells, and its genome does not integrate into the host cell genome, which reduces the risk of mutagenesis [61, 62]. From an industry perspective, replication-deficient HAd-5 gene therapy vectors can be upscaled, achieving high titres of up to 10^13^ viral particles (VPs)/mL following GMP.

However, HAd-5-based gene therapy products also carry a range of clinical challenges. Elimination of replication-deficient HAd-5 by the immune system poses a major challenge for clinical gene therapy trials when long-term transgene expression is required, for example, in the context of monogenic diseases. In contrast, immune-mediated elimination may not be an issue when acute/transient transgene overexpression is required, for example, in the context of promoting angiogenesis or triggering T-cell and humoral responses following vaccine administration. Based on the geographical setting, seroprevalence of neutralising antibodies (nAbs) for HAd-5 in humans ranges between 72 and 85.2% [63, 64]. Clinical trials have demonstrated that nAbs suppress the immunogenicity of HAd-5-based vaccine vectors, thereby hampering vaccine efficiency [65, 66]. Murine in vivo studies have identified the immunological basis of HAd-5 vector elimination, showing that systemic recombinant Ad delivery activated innate immune mechanisms which resulted in rapid Ad clearance and, hence, inefficient transgene delivery/expression [67, 68]. Yang et al. demonstrated that retrograde biliary E1-deleted HAd-5 delivery to mice not only resulted in desired hepatocyte transgene expression but also in low-grade viral gene expression [69]. This triggered a virus-specific cellular immune response culminating in the destruction of genetically modified hepatocytes highlighting the role of the adaptive immune system in eliminating recombinant HAd-5.

Targeted cardiovascular transgene delivery via the vascular route is further complicated by sequestration of replication-deficient HAd-5 in the liver, combined with reduced viral transduction efficiency in the vasculature and in aged cardiomyocytes [70–73]. More importantly, intraportal vein delivery of E1-deleted HAd-5 to non-human primates led to hepatotoxicity and features of potentially life-threatening systemic inflammatory response syndrome (SIRS) [74]. In the clinical context, intrahepatic artery delivery of E1-/E4-deleted HAd-5 harbouring the human *OTC* gene (encodes ornithine transcarbamylase) to an OTC-deficient patient triggered a fatal SIRS resulting in the termination of one of the first gene therapy trials in humans [75]. Finally, pre-clinical in vivo studies have demonstrated transient loss of first-generation HAd-5-mediated transgene expression due to episomal degradation [76, 77].

## Strategies to improve efficiency adenovirus-mediated transgene delivery

Local delivery of therapeutic HAds via intracoronary infusion, intramyocardial injection, and lumenal ex vivo saphenous vein graft (SVG) transduction provides local gene delivery, circumventing the need for intravenous systemic administration. In contrast, systemic HAd administration would be the preferred route for targeting vascular disorders such as PAH or atherosclerosis, or to provide potential for global delivery to an organ such as the heart that cannot be achieved through local gene transfer. Such approaches would be especially useful in applications where the transgene product is not secreted and acts inside the transduced cell. Whereas this strategy theoretically enables lumenal transgene delivery to diseased vasculature, it also risks deleterious side effects. In the context of VGF, efficient ex vivo SVG transduction requires high doses of HAd-5 due to low CAR presence on vascular smooth muscle cells (SMCs) and endothelial cells (ECs) risking toxicity and increased immune responses [78]. Strategies to improve target cardiovascular cell transduction and to reduce immunogenicity include the use of drug-mediated immunosuppression (reviewed in) [79], inhibition of coagulation factor (F)X carboxylation [80], and the use of genetically engineered chimeric HAds [78] and rare HAd serotypes [81].

## HAd capsid and fibre modifications

Genetic HAd capsid structure and fibre modification are strategies to alter cell/organ tropism and reduce immunogenicity. In addition to previously discussed findings, Parker et al. demonstrated that pseudotyping the HAd-5 capsid with both the fibre and penton of HAd-35 (HAd-5/F35/P35 chimaera) significantly improved luciferase transgene delivery to SVG SMCs compared to HAd-5/F35, HAd-35, and HAd-5 [78]. This effect was SMC-specific since transduction enhancement was not observed in SVG ECs. It appeared that pseudotyping the HAd-5 capsid with the HAd-35 penton base likely improved cell internalisation (compared to HAd-5) and intracellular trafficking (compared to HAd-35). Although HAd-5 transduction of whole SVGs remains inefficient at low viral doses, HAd-5 capsid integrity may be necessary for efficient transgene delivery to the nucleus in SVG SMCs [82].

The RGD-4C motif (tripeptide Arg-Gly-Asp) targets integrins [83] which HAd-5 utilises for internalisation [56]. Unlike CAR, the primary attachment receptor for HAd-5 and other species C HAds, integrins are expressed at high levels in vascular cells. To harness this entry mechanism, Work et al. inserted the RGD-4C motif into the HI loop (situated on the knob surface) of a replication-deficient HAd-5 expressing the *GFP* or luciferase transgenes [72]. HAd-5 RGD-4C-mediated transgene delivery to SVG SMCs, SVG ECs, and whole SVGs ex vivo was significantly increased compared to unaltered HAd-5, highlighting that this strategy is a viable approach to improve transgene delivery to SVGs during the CABG procedure. Furthermore, Nicklin et al. demonstrated that genetic incorporation of the EC-binding peptides SIGYPLP [71] (AdKO1SIG) and MSL and MTP [84] into the HI loop of replication-deficient HAd-5 s enabled selective transduction of vascular ECs.

Targeting HAd-5 to the vasculature following systemic administration is hampered by profound HAd-5 liver tropism [5]. Waddington et al. demonstrated that upon intravenous HAd-5 administration, FX binds to HAd-5 hexon proteins, thereby enabling hepatocyte transduction [85]. Alba et al. showed that inserting genetic mutations into the hypervariable region (HVR) of the HAd-5 hexon protein obstructed binding between FX and the modified hexon protein [86]. This resulted in reduced liver sequestration following intravenous administration to mice. Hence, a combination of RGD-4C insertion into the HI loop and preventing FX binding to the hexon protein appears to be a logical approach to redirect modified HAd-5 towards the vasculature. Robertson et al. generated a modified HAd-5 vector (HAd-5 T* HI loop) which demonstrated enhanced transgene delivery to SVG SMCs in vitro compared to unaltered HAd-5 [87]. In contrast, RGD-4C insertion did not prevent nAb neutralisation demonstrating that HAd-5 T* HI loop remained sensitive to immune attack. In addition, Ballmann et al. developed the novel chimeric HAd-20–42-42, a human Ad closely related to the human serotype 42 which contains the penton base of the human serotype 20 [88]. The authors demonstrated that HAd-20–42-42 showed a greater transduction potential of human saphenous vein ECs compared to HAd-5 and HAd-35 in the presence of FX. In vivo biodistribution studies in mice following systemic delivery revealed that HAd-20–42-42 mainly targeted the liver, lung, and spleen. Together, partial evasion of FX-dependent sequestration to the liver makes this vector an interesting candidate for systemic targeting of cardiovascular tissues.

In summary, HAd-5 capsid and fibre modifications are promising approaches to improve vascular cell transduction efficiencies and direct the modified HAd-5 vector towards the vasculature. Presented findings warrant future studies investigating safety and efficacy of local and systemic delivery of modified HAd-5-mediated transgene delivery in the context of vascular disease, although it is likely that tropism-modified adenoviral vectors may require bespoke GMP manufacturing processes to be developed to facilitate large-scale production of high-titre batches.

## Rare HAd serotypes

Human Ad-49 (HAd-49) belongs to the HAd subgroup D and utilises CAR, sialic acid, and cluster of differentiation (CD)46 receptors for cell attachment and internalisation [51]. E1/E3-deleted HAd-49-mediated *GFP* transgene delivery to primary SMCs and ECs from SVGs resulted in enhanced and more rapid transduction efficiency compared to HAd-5 GFP and suggests possible clinical relevance since the SVG only remains ex vivo for approximately 30 min during the CABG procedure [81]. Sera collected from 103 study patients did not yield any nAbs to HAd-49 compared to HAd-5. In addition, Bates et al. demonstrated that systemic delivery of HAd-49 to mice resulted in reduced liver sequestration while maintaining lung transduction indicating that this vector may be useful to deliver candidate therapeutic transgenes to the pulmonary vasculature and parenchyma [89]. Human Ad serotypes 35 (HAd-35) and 11 (HAd-11) belong to HAd subgroup B and utilise the CD46, CD80/86, receptor X, and HSPG receptors for cell entry [51]. Neutralising Abs to HAd-35 [90] and HAd-11 [91] demonstrated low seroprevalence in humans, and E1-deleted HAd-35- and HAd-11-mediated *GFP* transgene delivery to SVG SMCs was more efficient compared to HAd-5 GFP. In addition, intravenous HAd-35 delivery to mice did not result in liver sequestration compared to HAd-5 delivery [90]. Parker et al. confirmed findings from Vogels’s study demonstrating that HAd-35-mediated *GFP* delivery to SVG SMCs was more efficient compared to HAd-5 GFP [78].

Taken together, replication-deficient HAd-11, HAd-35, and HAd-49 appear to have the potential to efficiently deliver therapeutic transgenes to SVG SMCs in vitro and whole SVGs ex vivo. Low nAb seroprevalence to rare Ads in humans suggests that these serotypes may be able to evade the initial immune response following systemic administration. Hence, these serotypes may also represent as potential candidates for intravenous transgene delivery. Pre-clinical in vivo studies are warranted to investigate the efficacy and safety of local and systemic administration of rare HAd serotype-mediated transgene delivery in the context of experimental cardiovascular disease models to assess their ability to provide therapeutic levels of gene transfer in comparison to the widely used HAd-5 vector.

## Modulation of host immunity and inhibition of FX carboxylation

HAd-5-mediated gene therapy only enables transient transgene expression in host target cells. Hence, HAd-5 vector readministration may be necessary to sustain a continuous therapeutic effect in certain disease settings. However, the high prevalence of nAbs and immune system sensitisation results in rapid HAd-5 vector elimination which renders this strategy unviable. In order to overcome this barrier, host immunomodulation with immunosuppressive drugs and/or monoclonal antibodies may represent another strategy to circumvent immune-mediated elimination of adenoviral gene therapy vectors (reviewed in) [79]. Jooss et al. demonstrated that systemic co-administration of cyclophosphamide and Ad vectors blocked activation of CD4 + and CD8 + T cells as well as nAb formation resulting in prolonged transgene expression in the liver and lung of C57BL/6 mice [92]. In addition, a study by Lochmüller et al. found that administration of tacrolimus to adult dystrophic mice enabled prolonged Ad-mediated dystrophin expression by suppressing the humoral and cellular immune responses [93]. More recently, Leborgne et al. investigated the use of imlifidase in the context of adeno-associated virus (AAV)-mediated gene therapy [94]. Imlifidase is an endopeptidase which is capable of degrading circulating IgG and is being tested in transplant patients [95]. The authors showed that administration of imlifidase to mice and non-human primates was safe and enhanced AAV-mediated hepatic transgene delivery, even after readministration. In addition, the authors demonstrated that imlifidase reduced the amount of neutralising AAV antibodies in human sera in vitro.

Profound FX-mediated sequestration of HAd-5 to the liver presents another barrier to enabling efficient HAd-5-mediated gene transfer to non-liver targets following systemic administration. Waddington et al. pre-treated mice with warfarin, a known inhibitor of vitamin K-dependent carboxylation of FX, prior to systemic administration of recombinant HAd-5 [80]. The authors found that HAd-5 liver uptake was markedly reduced in warfarinised mice, indicating that warfarin may be a useful tool in detargeting HAd-5 from the liver. In addition, Schüttrumpf and colleagues investigated the effect of multiple anticoagulants on AAV- and Ad-mediated gene therapy [96]. The authors showed that administration of the FXa inhibitor tick anticoagulant peptide significantly reduced Ad-mediated liver uptake following systemic vector administration in C57BL/6 mice.

Together, these pre-clinical studies provide evidence that both immunomodulation and/or FX inhibition may represent attractive strategies to reduce host immune system-driven elimination and liver sequestration of systemically administered recombinant HAd-5 vectors. It is noteworthy that investigated immunosuppressive drugs and warfarin are already authorised treatments for humans, and hence, this may facilitate the design of future gene therapy trials in humans. Furthermore, it may be of interest to explore the effect of newer direct oral anticoagulants (FXa inhibitors), for example, apixaban, on liver sequestration of recombinant HAd-5 vectors in a pre-clinical setting.

## Industrial manufacturing of adenoviral vector-based products

Typical production volumes for Ad vectors to get into phase I–II clinical trials are in the range of 10–50 L and can be scaled up to even larger volumes when using suspension cells or high cell density packed-bed manufacturing systems like iCELLis or scale-X bioreactors [97]. When manufacturing in suspension, the typical cell seeding density lies between 0.5 and 1.0 million cells/mL resulting in approximately 10^13^ VPs per L for standard batch bioreactor processes [98, 99]. The latter is based on extensive data using the Ad5 vector, but yields with other serotypes are similar as packaging cells allow the formation of about 10^5^ VP per cell. A standard, six step vector purification process has been published previously consisting of (i) cell lysis and genomic DNA breakdown, (ii) clarification with dead end filters, (iii) concentration via ultrafiltration, (iv) anion exchange, (v) gel filtration, and (vi) dead-end filtration [100]. During the first step, detergent-based cell lysis increases the virus titre substantially by aiding the release of mature virions from the cell [98, 101–104]. However, this also increases the release of contaminants from the cells including intracellular proteins and genomic DNA. To rapidly reduce the nucleic acid chain lengths, an enzymatic digestion of DNA (using, e.g., DNase or Benzonase) is commonly used to reduce genomic DNA size to mere fragments [98, 105–107]. During the second step, a series of depth filters is used to remove cell debris and reduce bioburden load. The chosen depth filters typically allow the Ad vector to pass quickly while retaining contaminants [98, 107, 108]. A final 0.2-μm filter step using a sterile membrane allows for an “in-process” hold step which facilitates flexibility in the production planning. The third step involves product concentration mainly to reduce volume for the next purification steps. That said, this step typically also allows for a high reduction of small molecules such as small proteins. When using membranes up to 100 MDa, the virus with a molecular mass of around 170 MDa is concentrated, while small contaminants wash through [98, 107, 109, 110]. Step 4 involves an anion-exchange column purification as the Ad vector is negatively charged at physiological pH. Resins typically employed include Source-Q, Q-Sepharose-XL, or Fractogel DEAE [98, 99, 103, 106, 109–114]. This step typically results in a high recovery (between 60 and 90%) [106, 109, 111]. As the vector is eluted from the column by salt, the fifth purification step typically involves a gel filtration to remove the salt and to facilitate buffer exchange to final formulation. Resins used in this step are typically Toyopearl HW75F, Sepharex-G25 or Sephacryl-400HR [98, 99, 106, 113]. Again, high recoveries (up to 90%) of vector are readily reported [109]. The final step to produce a clinical-grade product is the filtration through a 0.2-μm sterilising grade filter. Typically, a purification process as described above results in an overall 60% product recovery with the product adhering to set regulations as stipulated in regulatory guidance documents (Ph. Eur 5.14, Ph. Eur 5.2.3, Ph. Eur 2.6.16, FDA1998, FDA 2010, EMA 2010) [100].

## Manufacturing challenges

### Cell and virus culture

Viral vectors for gene therapy are mainly produced using adherent cells for virus replication. However, adherent cells (e.g., HEK293) need a solid support for growth, and therefore, cell and subsequent virus culture scale-up is more challenging when compared with using suspension cells. Small-scale viral vector manufacturing has relied on different classical research laboratory methods using culture flask approaches, and traditional scale-up options have been stacked culture flasks [115, 116]. However, such horizontal scale-out allowing for the required surface for adherent cells to proliferate is cumbersome, often requires manual handling, may need open connections, and lacks monitoring and control for pH and dissolved oxygen [115, 117]. Therefore, the use of scalable fixed-bed bioreactors in viral vector clinical lot and commercial manufacturing has recently been explored successfully [97, 115, 118]. Fixed-bed bioreactor vessels offer a solid or porous support to immobilise adherent cells, in which the cells can be grown to high cell densities by perfusion of nutrients and oxygen. Such fixed-bed bioreactor vessels can support the manufacturing of batch sizes sufficiently large for clinical studies and for market introduction and can be either reusable or disposable [118]. In addition, with the increasing demand for viral vectors, it is anticipated that the development of a mix of alternative or additional production technologies in large-scale viral vector manufacturing: (i) suspension-based upstream processes in stirred-tank vessels, and (ii) a trend towards continuous bioprocessing approaches will be observed [117]. These various production technologies and modes of operation hold the promise of significantly increased batch sizes, therefore reducing manufacturing costs [117, 118].

## Virus purification

Third-generation HD Ad vectors remove all protein-coding sequences from the vector backbone, requiring these elements to be supplied in *trans*. Coinfection of cells with a HV (generally an empty first-generation vector) is thus necessary to produce these vectors [119, 120]. The gutless Ad vector not only eliminates the cellular immune responses against de novo synthesised viral proteins but also significantly expands its capacity for the transgene cassette [120]. Because production of a gutless vector requires helper functions from coinfected E1-deleted Ad, it is critical to remove the HV efficiently from the gutless vector preparations [97, 98]. Since the capsids of the HV and vector are indistinguishable, gradient ultracentrifugation is the currently available method to remove HV [119]. However, ultracentrifugation is most often used in pre-clinical development [121]; the method is expensive and considered not convenient for large-scale manufacturing following GMP requirements [97, 122]. Therefore, alternatives like sequential use of anion-exchange columns and density gradients have been explored to improve separation and enrichment of full vector particles [97, 122]. In addition, a combination of chromatographic methods based on capture antibodies, ionic exchange, size exclusion, hydrophobic interaction, and immobilised metal affinity columns has been described as a feasible alternative [97, 122].

## Complementing cell line development

Efforts to generate a cell line to complement these vectors initially failed, likely due to the inherent cytotoxicity of some Ad proteins [119]. However, recently successful production of HD-Ad in the absence of a HV, using a helper plasmid instead, was reported [123]. Utilising this helper plasmid, large quantities of recombinant HD Ad were successfully produced. Importantly, the helper plasmid-based system exclusively produced recombinant HD Ad with no generation of helper plasmid-originating Ad and replication-competent Ad [123].

## Good manufacturing practices

Ultimately, the choice of cell lines and production equipment and methods for large-scale manufacturing of clinical-grade viral vectors requires a substantial investment in time and capital as each system requires deliberate and careful optimisation and validation, in compliance with regulatory requirements and current GMP [116, 121, 124]. For example, in large-scale manufacturing of the first approved oncology gene therapy product Gendicine® (HAd-5 expressing p53), a proprietary perfusion-based bioreactor system was used for cell and virus culture, and commercial equipment and conventional methods (membrane filtration, ultrafiltration, and chromatography) were applied in purification [125]. The chemistry, manufacturing, and controls (CMC) for Gendicine® were established to be fully compliant with investigational new drug (IND) application and current GMP requirements for both the Chinese and US FDA. The critical components, such as master cell and vector banks, working cell and vector banks, and other raw materials, were all analysed according to robust assay panels [125]. The major in-process testing items were vector infectivity and validity, vector quantity in the crude harvest, vector purity, and impurity analysis. The main lot release testing parameters were sterility, vector purity, concentration, VP measurement, infectivity/plaque forming unit (PFU), potency (p53 gene expression/bioactivity), replication-competent Ad, AAV, residuals (host cell DNA, protein, bovine serum albumin), endotoxin, and mycoplasma [125].

## Regulatory requirements

Gene therapy medicinal products are innovative and promising treatment strategies that seek to modify or manipulate the expression of a gene or to alter the biological properties of living cells for therapeutic use (a list of authorised viral vector-based GTMPs with their respective regulatory guidance is provided in Table 2). As a novel class of therapeutics, GTMPs are faced with particular challenges when it comes to regulatory assessment. GTMPs are complex pharmaceuticals, often requiring specific administration modalities, and with specific safety issues linked to their use such as integrational mutagenesis and off-target biodistribution. Manufacturing considerations for GTMPs include product quality, purity, potency, and the need to meet stringent GMP requirements (as discussed above [126]). Recognising these challenges, regulatory agencies have developed specific frameworks to deal with the assessment of GTMPs. In Europe, the EMA has provided recommendations regarding CMC information that needs to be submitted in a GTMP IND application to meet local legal requirements (for EU: Directive 2001/83/EC code relating to medicinal products for human use, Regulation (EC) No. 1394/2007 on advanced therapy medicinal products, and Directive 2001/18/EC for deliberate release of genetically modified organisms, while other member states consider contained use according to Directive 2009/41/EC). The purpose of such guidance is to inform how to provide sufficient CMC information required to assure product safety, identity, quality, purity, and strength (including potency) of the investigational product. In the USA, the FDA has developed similar guidance documents on topics relevant to GTMPs, including CMC information for gene therapy INDs [127], long-term follow-up after administration of GTMPs [128], and guidance on specific indications (e.g. haemophilia, retinal disorders, and neurodegenerative diseases). In Europe, the Committee for Advanced Therapies (CAT) is responsible for making marketing authorisation recommendations to the Committee of Human Medicinal Products (CHMP) [129]. A recent review identified major objections, issues, and concerns raised during the marketing authorisation application (MAA) process for products resulting from the interaction of both committees between 2008 and 2017 [130]. During the first few years following CAT establishment, the quality issues were often identified as major deficiencies. Regulators frequently identified problems with the production process, drug specification, or release assay data, perhaps linked to the fact that the development of many GTMPs during that period was initiated by academic institutions, which may not have had the appropriate resources or training to adequately anticipate quality issues. Issues at the non-clinical level appeared to be less frequent and were mainly focused on pharmacokinetics and pharmacodynamics. Of particular relevance to this review, regulators have raised concerns regarding the persistence of replication-competent adenovirus (RCA) particles in GTMP batches. Conversely, clinical efficacy and safety issues appeared to have a major role in unsuccessful MAA outcome for GTMPs [130]. Regulators have criticised GTMPs for a lack of clinical efficacy, changing or using non-validated primary endpoints, and the application of post-hoc and subgroup analyses. Particular concerns have been raised regarding the risk of immunogenicity, mediated by humoral and cellular responses targeting the GTMP vectors and/or transgenes.

**Table 2:** List of approved virus-based GTMPs with regulatory guidance

Brand name	Product name	Indication	Viral vector base	Company	Regulatory product number
Oncorine	rAd5-H101	Nasopharyngeal carcinoma	HAd-5	Shanghai Sunway Biotech Co., Ltd	CFDA
Gendicine	rAd-p53	Head and neck squamous cell carcinoma	HAd-5	Shenzhen SiBiono GenTech Co., Ltd	CFDA
Imlygic	Talimogene laherparepvec	Melanoma	HSV1	Amgen Europe B.V	EMA: EMEA/H/C/002771 FDA: STN: 125,518
Yescarta	Axicabtagene ciloleucel	Lymphoma	LV	Kite Pharma EU B.V	EMA: EMEA/H/C/004480 FDA: STN: BL 125,643
Tecartus	Brexucabtagene autoleucel	Mantle cell lymphoma ALL	LV	Kite Pharma EU B.V	EMA: EMEA/H/C/005102 FDA: STN: BL 125,703
	Etranacogene dezaparvovec	Haemophilia B	AAV5	uniQure Biopharma B.V	EMA: EU/3/18/1999
Luxturna	Voretigene neparvovec	Leber’s congenital amaurosis	AAV2	Novartis Europharm Limited	EMA: EMEA/H/C/004451 FDA: STN: 125,610
Zolgensma	Onasemnogene abeparvovec	Spinal muscular atrophy	AAV9	Novartis Gene Therapies EU Limited	EMA: EMEA/H/C/004750 FDA: STN: 125,694
Kymriah	Tisagenlecleucel	Large B-cell lymphoma B-cell precursor acute lymphoblastic leukaemia	LV	Novartis Europharm Limited	EMA: EMEA/H/C/004090 FDA: STN: 125,646
Zynteglo	Betibeglogene autotemcel	Transfusion-dependent beta-thalassemia	LV	Bluebird bio B.V	EMA: EMEA/H/C/003691
Strimvelis		Severe combined immunodeficiency	LV	Orchard Therapeutics B.V	EMA: EMEA/H/C/003854

*r* recombinant *HAd-5* human adenovirus serotype 5, *AAV* adeno-associated virus, *LV* lentivirus, *HSV1* herpes simplex virus 1, *ALL *acute lymphoblastic leukaemia, *EMA* European Medicines Agency, *CFDA* China Food and Drug Administration, *FDA* US Food and Drug Administration

## Adeno-associated and lentiviral vectors: an alternative to adenoviral vectors in cardiovascular gene therapy

It is important to highlight that other viral vectors have been investigated for cardiovascular gene therapy applications. In particular. adeno-associated virus (AAV) and lentivirus (LV)-based gene therapy vectors represent alternative platforms for delivering therapeutic transgenes to target cardiovascular tissues (reviewed in) [131]. Especially in the case of AAV vectors, there has been significant translation into clinical trials for cardiovascular gene therapy, and therefore, these vectors are briefly introduced here to aid as a comparator to developments using adenoviral vectors which are then outlined below. A side by side comparison including advantages and disadvantages of Ad-, AAV-, and LV-based gene therapy vectors is presented in Table 1.

AAVs are replication-defective, non-enveloped viruses which belong to the family of Parvoviridae (reviewed in) [132]. An icosahedral capsid protects single-stranded (ss) AAV DNA (~ 4.7 kb) which is made up of the three genes *Rep* (replication), *Cap* (capsid), and *Aap* (assembly) flanked by two ITRs (reviewed in) [133]. Replication of wild-type AAVs depends on co-infection with a HV, for example, an AdV. In AAV-based gene therapy vectors, the *Rep*, *Cap*, and *Aap* genes are substituted with a transgene of interest. Given the absolute lack of viral DNA, recombinant AAVs are in essence engineered replication-deficient nanoparticles capable of delivering transgenes of interest to target cells. Following delivery, the transgene is stably expressed in the nucleus and remains extrachromosomal. *Rep* gene absence in recombinant AAVs hinders site-specific integration into chromosome 19 (reviewed in) [134]. Whereas AdV-mediated transgene expression is transient in nature, AAV-based gene therapy enables stable and long-term transgene expression in target host cells. Approved AAV-based GTMPs include Luxturna for treating Leber’s congenital amaurosis [135] and Zolgensma for treating spinal muscular atrophy [136] (Table 2).

Lentiviruses are spherical, enveloped, ssRNA viruses which belong to the genus of the Retroviridae family [137]. The lentiviral capsid contains two sense-strand RNAs (~ 9 kb) as well as reverse transcriptase, integrase, and proteinase proteins (reviewed in) [131]. Depending on their genome organisation, LVs are either classified as simple or complex viruses. The human immunodeficiency virus (HIV)-1 is a complex virus, and many LV vector systems are derived from HIV-1. To date, there are three generations of replication-deficient HIV-1-based gene therapy vectors. Following infection and reverse transcription, LV DNA non-randomly and preferentially integrates into the host cell genome at transcriptionally active sites (reviewed in) [138]. Hence, LV-based gene therapy enables stable and long-term transgene expression in target host cells. However, compared to Ad- and AAV-based vector systems, recombinant LV-mediated transgene delivery poses a risk of mutagenesis second to its ability to integrate into the host cell genome. Approved LV-based GTMPs include Kymriah for treating large B-cell lymphoma and B-cell precursor acute lymphoblastic leukaemia as well as Zynteglo for treating transfusion-dependent beta-thalassemia (Table 2).

## Adenovirus-based gene therapies for cardiovascular diseases

Current cardiovascular gene therapy trials in humans focus on improving cardiac angiogenesis and function. Studies in humans found that local delivery of recombinant first-generation HAd-5 to the heart was generally safe in the short term [2, 13–15, 139]. However, it must be pointed out that only a limited number of cardiac gene therapy trials in humans have been carried out so far and that the number of participants per study has always been low. Hence, many more studies investigating larger participant sizes are necessary to determine long-term safety and efficacy of HAd-5-based gene therapies. A list of current and future cardiovascular gene therapy trials is provided in Table 3.

**Table 3:** List of current cardiovascular gene therapy trials employing recombinant HAd-5 vectors

Trial name	Start and finish date	Indication	Product name	Viral vector, delivery route and viral particles	Number of patients	Phase	Clinical trial number	Company/university
AdeLE	06/2018 until 12/2024	Secondary lymphoedema	Lymfactin^®^	AdAptVEGF-C Ex vivo perinodal injection 1 × 10^11^ VPs	39	II	NCT03658967	Herantis Pharma Plc
	06/2016 until 02/2024	Secondary lymphoedema	Lymfactin^®^	AdAptVEGF-C Ex vivo perinodal injection 1 × 10^10^ and 1 × 10^11^ VPs	15	I	NCT02994771	Herantis Pharma Plc
EXACT	01/2020 until 09/2021	Drug-refractory CAD	XC001	AdVEGFXC1 Transthoracic epicardial intramyocardial injection 1 × 10^9^, 1 × 10^10^, and 1 × 10^11^ VPs	29	I/II	NCT04125732	XyloCor Therapeutics Inc
	12/2020 until 10/2030	Drug-refractory CAD		AdVEGF-AII6A + Transthoracic epicardial intramyocardial injection 1 × 10^8^, 1 × 10^9^, and 1 × 10^10^ VPs	41	I/II	NCT01757223	Weill Medical College of Cornell University
FLOURISH^1^	06/2019 until 06/2023	Heart failure with reserved ejection fraction	RT-100	Ad5.hAC6 Intracoronary injection No information available yet	0	III	NCT03360448	Renova Therapeutics
	07/2010 until 11/2017	Congestive heart failure	RT-100	Ad5.hAC6 Intracoronary injection 3.2 × 10^9^ to 10^12^ VPs	56	I/II	NCT00787059	Renova Therapeutics
AFFIRM	06/2021 until 12/2022	Refractory angina due to myocardial ischaemia	Generx^®^ (Alferminogene tadenovec)	Ad5FGF-4 Intracoronary infusion under transient ischaemia 6 × 10^9^ VPs	160	III	NCT02928094	Angionetics Inc., Huapont Life Sciences
	04/2018	Ischaemic heart disease		Ad-HGF Trans-endocardial injections	30	IIa	n/a	The First Affiliated Hospital with Nanjing Medical University
	07/2017 (3-year study)	Severe CAD		Ad-HGF Intracoronary infusion 5 × 10^9^, 1 × 10^10^, and 2 × 10^10^ PFUs	22	I	n/a	The First Affiliated Hospital with Nanjing Medical University
	07/2008 (35-day and 11–14 month follow ups)	3-vessel CAD		Ad-HGF Intracoronary infusion 5 × 10^9^, 1 × 10^10^, and 2 × 10^10^ PFUs	18	I	n/a	The First Affiliated Hospital with Nanjing Medical University

*CAD* coronary artery disease, *VEGF* vascular endothelial growth factor, *VPs* viral particles, *AC6* adenylyl cyclase 6, *FGF4* fibroblast growth factor 4, *HGF* hepatocyte growth factor, *PFU* plaque forming unit

^1^Currently withdrawn due to re-evaluation of clinical development and strategy

## Drug refractory angina pectoris due to obstructive coronary artery disease and post-myocardial infarction

Refractory angina pectoris (AP) is a chronic chest pain condition (> 3 months) caused by reversible myocardial ischaemia against the background of coronary artery disease (CAD) which cannot be controlled with drug or revascularisation therapy [140]. In Europe, refractory AP cases range between 30 and 50 thousand patients per year [140]. Several gene therapy trials have been conducted or are planned for patients with drug refractory AP and obstructive CAD who are unsuitable for standard revascularisation therapies. The rationale is to improve cardiac blood flow/oxygen supply by delivering therapeutic transgenes which promote myocardial angiogenesis.

In 1998, Mack and colleagues investigated the effect of direct intramyocardial injection of AdGVVEGF121.10 (replication-deficient HAd-5 expressing the pro-angiogenic VEGF 121 isoform, 1 × 10^8^ PFU/site) via thoracotomy on myocardial ischaemia in pigs [141]. The study revealed that AdGVVEGF121.10 lead to an increase in myocardial angiogenesis, perfusion, and function following experimental myocardial infarction paving the way for gene therapy trials in humans. In 1999, a phase I trial (*N* = 21 CAD patients) suggested that direct intramyocardial injection of AdGVVEGF121.10 (total doses 4 × 10^8^, 10^8.5^, 10^9^, 10^9.5^, and 10^10^ particle units) during coronary artery bypass graft (CABG) surgery or minithoracotomy drove myocardial angiogenesis (determined by coronary angiography) and improved ventricular function (determined by stress sestamibi scan) in the area of vector administration [14]. Encouragingly, there was no evidence of vector-related adverse events at 6-month follow-up [15]. The larger REVASC trial (*N* = 67) investigated the effect of direct intramyocardial delivery of AdVEGF121 (replication-deficient HAd-5 expressing only the VEGF 121 isoform) via mini-thoracotomy versus maximum medical treatment in patients with clinically significant CAD and no conventional option for revascularisation [13]. The investigators found that AdVEGF121 delivery to 30 intramyocardial sites of the left ventricle (total dose 4 × 10^10^ particle units) resulted in improved exercise-induced ischaemia compared to continued maximum medical treatment. Post-mortem analysis of one patient who died from cardiogenic shock second to a peri-operative myocardial infarct for 19 days following AdVEGF121 administration revealed robust neovascularisation around the injection sites, providing some evidence for proof of concept in humans. However, nuclear perfusion imaging did not reveal an increase in myocardial perfusion in AdVEGF121-treated patients. Encouragingly, the number of adverse of events, apart from procedure-related events in the thoracotomy group, was not significantly different indicating that cardiac gene therapy was at least safe in the short term.

However, there remains concern over potential unfavourable safety characteristics of the VEGF isoform 121. This includes induction of local oedema [142] and pro-oncogenic properties [143]. Given the fact that longer VEGF isoforms demonstrate a more favourable safety profile [143], Amano et al. developed two novel replication HAd-5 vectors which expressed all three pro-angiogenic VEGF isoforms 121, 165, and 189 (AdVEGF-All and AdVEGF-All6A +) [8]. Although both vectors encode isoform 121, alteration of splicing sequences for exon 6A in AdVEGF-All6A + promote the expression of the less pro-oncogenic isoform 189 over 121. Intramuscular injection of both AdVEGF-A11 (1 × 10^5^ particle units) and AdVEGF-A116A + (1 × 10^5^ particle units) into the hind limb of rats prior to excision of the external iliac artery resulted in a similar induction of muscular angiogenesis and increase in blood flow recovery compared to AdNull-treated rats. Importantly, intravenous and intratracheal administration of AdVEGF-A116A + resulted in reduced tumour growth and pulmonary oedema compared to AdVEGF-A11 indicating a better safety profile. Mathison and colleagues went on to show that direct injection of AdVEGF-A116A + (total dose 1 × 10^9^) around infarcted myocardium in mice resulted in enhanced myocardial angiogenesis and improved left ventricular function compared to AdNull-treated animals [144]. Moreover, Kaminsky et al. went on to show that direct administration of AdVEGF-A116A + (1 × 10^5^, 10^6^, and 10^7^ particle units) to ischaemic myocardium of rats was safe and did not lead to any off-target side effects [145]. This pre-clinical work has paved the way for two phase I/II trials which aim to investigate the safety and efficacy of direct epicardial AdVEGF-All6A + (XC001) delivery to ischaemic myocardium via minimally invasive transthoracic injection. The EXACT trial is sponsored by XyloCor Therapeutics Inc. (PA, USA), and the second trial (NCT01757223) is sponsored by Weill Medical College of Cornell University (NY, USA). Whereas the shorter EXACT trial will primarily investigate patient safety, the 10-year study sponsored by Weill Medical College will investigate change in exercise tolerance, segmental wall motion in treated territories by echocardiography, and segmental wall motion/perfusion in treated territories by MRI and AP occurrence in AdVEGF-All6A + and AdNull (placebo)-treated trial patients.

Intracoronary artery delivery of HAd-5 vectors represents another delivery strategy for cardiac gene therapy. Gao et al. investigated the effect of intracoronary artery delivery of Ad5FGF4 (replication-deficient HAd-5 expressing pro-angiogenetic fibroblast growth factor (FGF)4) on myocardial ischaemia in pigs [146]. The authors found that Ad5FGF4 treatment ameliorated regional-induced ventricular dysfunction and perfusion and this persisted for at least 12 weeks. Importantly, transgene FGF4 protein was only detected in hearts and not in any extracardiac sites. Furthermore, histologic examination of all organs did not reveal any abnormal findings, and FGF4 remained undetectable in plasma samples following intracoronary artery delivery of Ad5FGF4. Although no extracardiac abnormalities were detected in this study, a long-term safety concern regarding pro-oncogenic properties of FGF4 remains [147].

The AGENT clinical trials in the early 2000s investigated safety and efficacy of intracoronary delivery of Ad5FGF4 (Generx®, alferminogene tadenovec) in patients with chronic stable angina [16, 17] (AGENT3: 3.3 × 10^8^, 10^9^, 10^10^ VPs and 1.0 × 10^8^, 10^9^, 10^10^; AGENT4: 1.0 × 10^10^ VPs). In general, Ad5FGF4 treatment was safe with only some patients developing transient deranged liver function tests, thrombocytopenia, and elevated temperatures. Importantly, no significant increase in adverse events was noted in the Ad5FGF4 versus placebo arm at 12-month follow-up. Encouragingly, pooled subgroup analysis of the AGENT3 and 4 trials revealed an improvement in exercise tolerance time, time to angina, time to 1 mm ST-segment depression, and the Canadian Cardiovascular Society class in female study participants who received 1.0 × 10^10^ VPs of Ad5FGF4 indicating a gender-dependent effect [148]. Myocardial perfusion determined by single-photon emission computed tomography (SPECT) in the AGENT4 trial only demonstrated a non-significant trend towards improvement following AdFGF4 treatment versus placebo. Limited efficacy of Ad5FGF4 treatment may be explained by inefficient transgene delivery to diseased cardiac tissue in this clinical setting. To further improve HAd-5-mediated transgene delivery to cardiac tissue, recent efforts have focused on optimising the administration methods for delivery. A trial in pigs demonstrated that transgene delivery was improved by ischaemic reperfusion achieved by two consecutive catheter balloon-mediated coronary artery occlusions followed by intracoronary nitroglycerine administration [149]. The ASPIRE trial confirmed the safe use of this technique to deliver Ad5FGF4 to 11 human trial participants [150]. The phase III AFFIRM study sponsored by Angionetics Inc. (CA, USA) is investigating Ad5FGF4 safety and efficacy in 160 trial participants following intracoronary delivery under transient ischaemia. The study is expected to finish at the end of 2022.

In a phase I trial, Yang et al. investigated the safety of intracoronary delivery of Ad-HGF (replication-deficient HAd-5 expressing the pro-angiogenic growth factor human hepatocyte growth factor; 5 × 10^9^, 1 × 10^10^, and 2 × 10^10^ PFU) in patients with severe CAD [151]. Importantly, the authors did not detect any serious adverse events (SAEs) at 11–14 months of follow-up indicating that intracoronary delivery of Ad-HGF was at least safe in the short term. Building on this study, a phase IIa trial (*N* = 30 patients) investigated the safety and efficacy of percutaneous endocardial injection of Ad-HGF in patients with post-MI heart failure [139]. The authors did not note any SAEs and were able to demonstrate a significant improvement in left ventricular end-diastolic dimension and left ventricular ejection fraction (LVEF) at 6-month follow-up. Findings from these 2 small studies are encouraging; however, as with VEGF and FGF4, a long-term safety concern remains given strong pro-oncogenic properties of HGF [152].

## Heart failure with reduced ejection fraction

Heart failure results from the inability of the heart to pump a sufficient amount of blood at normal filling pressures to organs around the body. Typical symptoms include breathlessness, fatigue, exercise intolerance, and ankle swelling reducing quality of life [153]. Based on LVEF, HF is further stratified into HF with reduced ejection fraction (HFrEF, LVEF < 40%), HF with mildly reduced ejection fraction (HFmrEF, LVEF 40–49%), and HF with preserved ejection fraction (HFpEF, LVEF > 50%) [154].

Since morbidity and mortality of HFrEF have remained high, gene therapy is an attractive novel approach to restore ventricular function. Restoring impaired sarco/endoplasmic reticulum Ca^2+^-ATPase (SERCA2A) expression, which regulates Ca^2+^, in patients with HFrEF represents one treatment strategy. Kawase et al. demonstrated that intracoronary delivery of recombinant AAV-1/SERCA2A (1 × 10^12^ VPs) to pigs with volume overload HF was well tolerated, safe, reversed systolic dysfunction, and improved ventricular remodelling [155]. Importantly, the investigators showed that cardiac *SERCA2A* expression levels were significantly higher in AAV-1/SERCA2A-treated pigs compared to control groups at 2-month follow-up providing evidence for stable long-term transgene expression. A subsequent phase II clinical trial (CUPID-1, *N* = 39 patients) investigated safety and efficacy of intracoronary delivery of AAV-1/SERCA2A (6 × 10^11^, 3 × 10^12^, and 1 × 10^13^ VPs) to patients with advanced HF [156]. At 6 and 12 months, the high-dose group (1 × 10^13^ VPs) versus placebo demonstrated an improvement or stabilisation of the New York Heart Association (NYHA) class, Minnesota Living With Heart Failure Questionnaire, 6-min walk test, peak maximum oxygen consumption, N-terminal prohormone brain natriuretic peptide levels, and LV end-systolic volume as well as a significant increase in time to clinical events and a decrease in frequency of cardiovascular events. Importantly, no SAEs were detected. Zsebo et al. went on to show that the risk of prespecified recurrent cardiovascular events was reduced by 82% in the high-dose versus placebo group at 3-year follow-up [157]. The authors also provided evidence of long-term cardiac *SERCA2A* expression in a total of 3 patients (highest dose group) at 22, 23, and 31 months, respectively. Cardiac samples were obtained from deceased patients or patients undergoing surgery (e.g., heart transplantation). Of note, no safety concerns were noted at 3-year follow-up. Surprisingly and disappointingly, the ensuing much larger CUPID-2 trial (*N* = 250 patients) showed that intracoronary delivery of AAV-1 SERCA2A (1 × 10^13^ VPs) did not improve the clinical course of patients with HFrEF [3]. No significant increase in SAEs was recorded in the treatment arm.

It is not fully understood why the CUPID-2 trial failed to replicate CUPID-1 findings. The authors were able to obtain cardiac samples from 7 patients (either deceased or requiring surgical intervention) in which they were able to show low levels of vector DNA (range: < 10–192 DNA copies/μg). This was comparable to vector DNA levels obtained in CUPID-1 (range: < 20–561 DNA copies/μg). Furthermore, study participants who were positive or equivocal 1:2 for AAV-1 nAbs were excluded from the study avoiding humoral immune-driven clearance following intracoronary vector administration. Although the investigators detected high expected AAV-1 nAb seroconversion rates following vector administration, these nAbs would unlikely explain low DNA vector expression levels since the Ab response only occurs after days to weeks. In addition, anti-AAV-1-specific CD8 + T-cell responses were mostly negative excluding cellular immune-driven clearance. One potential reason for the absence of gene therapy efficacy may be explained by differences in AAV-1 preparations between CUPID-1 and CUPID-2. The authors found that AAV-1/SERCA2A doses given to patients in CUPID-1 contained 85% of empty AAV-1 capsids (absence of ss *SERCA2A* DNA) compared to 25% of empty capsids in CUPID-2. The authors hypothesised that a higher proportion of empty AAV-1 capsids in CUPID-1 may have acted as potential decoys capable of blocking inhibitory Abs as well as other interfering serum substances, therefore hindering immune-driven degradation of therapeutic AAV-1 particles [158]. As with the HAd-5-based AFFIRM or upcoming FLOURISH trials, it may be speculated that introducing transient myocardial ischaemia ± intracoronary nitroprusside delivery following vector delivery may improve AAV-mediated transgene delivery to cardiac tissue. Despite the advantage of AAV-mediated stable long-term transgene expression, which is desirable in a cardiac disease setting, the absence of treatment efficacy in CUPID-2 demonstrates a need for alternative vector systems such as HAd-5.

Adenylyl cyclase (AC)6 contributes to efficient cardiac function through converting ATP to the cyclic (c)AMP [2]. Lai et al. showed that intracoronary injection of Ad5.hAC6 (1.4 × 10^12^ VPs, followed by nitroprusside) in pigs with pacemaker-induced LV dysfunction resulted in short-term improvement in LV function and a reduction in LV dilatation [159]. Importantly, the authors demonstrated cardiac AC6 transgene expression and LV cAMP generation following Ad5.hAC6 administration indicating successful transgene transfer. A subsequent phase I/II trial in patients with HFrEF (*N* = 56) confirmed the short-term safety of intracoronary Ad5.hAC6 (3.2 × 10^9^, 3.2 × 10^10^, 1 × 10^10^, 3.2 × 10^11^, and 1 × 10^12^ VPs)/nitroprusside delivery with no significant increase in SAEs noted in the treatment arm at approximately 1-year follow-up [2]. The authors revealed that only participants with non-ischaemic HFrEF who received 3.2 × 10^11^ and 1 × 10^12^ VPs demonstrated sustained increases in LVEF at 12-week follow-up compared to participants with ischaemic HFrEF receiving the same doses as well as all placebo groups. In contrast, the HF hospital admission rate was not significantly reduced in Ad5.hAC6 trial participants compared to placebo. Given the fact that this trial only demonstrated modest Ad5.hAC6 treatment effects in a subset of patients, it may be speculated that transgene delivery and uptake may have been insufficient. Since this study did not screen study participants for HAd-5 nAbs prior to randomisation and was unable obtain cardiac tissue for confirmation of hAC6 expression, it is difficult to comment on potential immune-driven vector elimination and/or low vector transduction efficiency in cardiac tissue. Furthermore, given the fact that HAd-5 only enables transient transgene expression, it is likely that even if a short-term therapeutic effect is observed in a cardiac disease setting, such as HFrEF, repeat intracoronary injections of HAd-5 expressing non-oncogenic therapeutic transgenes may be necessary to ensure a continuous therapeutic effect. In this hypothetical scenario, it is likely that repeat immune system exposure to HAd-5 would result in an increase in nAb formation culminating in rapid vector elimination and, hence, in insufficient transgene transfer. Potential strategies to overcome this barrier will be addressed later in this review. Nevertheless, Renova™ Therapeutics (CA, USA) is sponsoring the upcoming phase III FLOURISH trial which aims to investigate safety and efficacy of intracoronary Ad5.hAC6 (RT-100)/nitroprusside delivery to HFrEF patients [160]. Clinical endpoints will determine HF hospitalisation rates, adverse clinical event rates, and changes in EF.

## Heart failure with preserved ejection fraction

HFpEF is diagnosed in patients with signs or symptoms of HF, normal or mildly abnormal systolic LV function (LVEF > 50), and evidence of diastolic LV dysfunction [161]. Structural alterations include cardiomyocyte hypertrophy, interstitial fibrosis, and capillary rarefaction [162]. Several community-based cohorts reported that HFpEF accounts for a large population (51–63%) of HF cases in the USA and Europe (reviewed in) [163]. Risk factors for HFpEF/HFmrEF include age, female gender, systemic hypertension, obesity, and type 2 diabetes [164]. Whereas treatment with ACE inhibitors, mineralocorticoid receptor antagonists, β-blockers, and diuretics improves survival of HFrEF patients, these drugs do not demonstrate a survival benefit for HFpEF or HFmrEF patients [153]. Since global societies are growing older and HFpEF risk factors are becoming more prevalent, the incidence of HFpEF is set to increase in the future. Together, these facts highlight the need to develop novel efficacious therapies for HFpEF patients.

It is noteworthy that pre-clinical HFpEF and HFrEF research is challenging since commonly available murine HF models do not fully recapitulate human pathology [165]. Given the favourable characteristic of stable long-term transgene expression, AAV vectors have been favoured over Ad vectors in the pre-clinical development of gene therapies for cardiac diseases.[166–168]. Hence, only a limited number of studies investigated the use of Ad in diastolic/HFpEF mouse models. Shuai et al. demonstrated that intramyocardial injection of an E1/E3-deleted HAd-5 expressing human *RLN2* (encodes relaxin-2) improved diastolic dysfunction, a key feature of HFpEF, in a rat model of HF [169]. It is hypothesised that cardiac capillary rarefaction is a driver of HFpEF and, hence, in theory a point could be made that promoting angiogenesis may be a strategy to counter progression of HFpEF. In this case, one may argue that using an HAd-5 approach to deliver a pro-angiogenetic transgene may be more favourable than an AAV or LV approach based on transient transgene expression which would be favourable given potential pro-oncogenic properties of pro-angiogenic proteins such as VEGF, FGF4, or HGF. Encouragingly, Schiatterella et al. have developed a novel murine HFpEF model which more accurately mimics human disease [170].

## Vein graft failure following coronary artery bypass graft surgery

Coronary artery bypass graft (CABG) surgery is a surgical revascularisation technique which commonly utilises the great saphenous vein to bypass a critically stenosed coronary artery to re-establish blood flow to cardiac tissue [171]. Within the first year following CABG surgery, 10–15% of saphenous vein grafts (SVGs) occlude, and approximately 50% fail after 10 years [172]. Vein graft failure is driven by early thrombosis, occlusive neointima formation (NF), and accelerated atherosclerosis. Although pharmacological single antiplatelet [173] and lipid lowering [174] therapies reduce VGF rates, there remains an unmet clinical need to improve long-term SVG outcomes.

Pre-clinical studies have shown that gene therapy is an attractive strategy to prevent experimental NF in rodent vascular injury models [175–178]. From a translational point of view, lumenal Ad-mediated transgene delivery to human SVGs ex vivo pre-implantation may be achieved [81], thereby eliminating off-target effects. George et al. showed that RAdTIMP-3 (E1-deleted HAd-5 expressing tissue inhibitor of metalloproteinase-3 from a CMV promoter) inhibited NF in ex vivo human pre-implantation SVG organ cultures and in porcine SVGs for 28 days following carotid artery interposition grafting, a translationally relevant large animal surgical model [179]. TIMP3 inhibits matrix metalloproteinase activity and is pro-apoptotic in vascular smooth muscle cells leading to inhibition of NF [179, 180]. A subsequent study by George et al. confirmed that ex vivo RAdTIMP-3 delivery to porcine SVGs reduced NF 3 months after carotid artery interposition graft procedures [9]. This is a significant observation with the knowledge that transgene expression following first-generation HAd-5 delivery is typically lost by 28 days due to the host immune response [69] and suggests that early and acute intervention to prevent NF might be sustained. In parallel to the development of HAd-based gene therapy for VGF, the PREVENT trials (Project of Ex-Vivo Vein Graft Engineering via Transfection) provided the first proof of concept for ex vivo delivery of a the none viral-based gene therapy to SVGs in human CABG patients [181, 182]. Despite showing favourable results in the PREVENT II trial (*N* = 200 CABG patients), the larger PREVENT IV trial (*N* = 3014 CABG patients) revealed that ex vivo delivery of edifoligide, an oligodeoxynucleotide decoy known to block vSMC proliferation, did not significantly impact on SVG restenosis rates at early time points (12–18 months) [182] and at 5 years [181]. It is noteworthy, that although there is no clinical data available to support this hypothesis, it is likely that delivery efficiency and subsequent efficacy of a therapeutic agent is much higher when using Ad-based gene therapy compared to employing oligodeoxynucleotide decoys in SVGs. Overall, HAd-5-based gene therapy strategies warrant future clinical trials investigating VGF in CABG patients.

## Hereditary pulmonary arterial hypertension

Hereditary PAH (HPAH) is a rare genetic class I PAH disorder which causes abnormal pressure elevation (> 25 mmHg) within the pulmonary vasculature resulting in impaired blood oxygenation, breathlessness, impaired exercise tolerance, and right heart strain [183, 184]. Disease management focuses on symptom relief including oxygen supplementation, diuretics for right HF, calcium channel blockers, phosphodiesterase inhibitors, endothelin receptor antagonists, guanylate cyclase stimulators, prostacyclin analogues, and prostacyclin receptor agonists [183]. Apart from lung transplantation, no curative therapies are available for HPAH patients to date. Approximately 75% of HPAH patients harbour mutations within the *BMPR2* gene (encodes bone morphogenetic protein receptor 2) [185] shown to drive disease onset and progression [186]. Hence, targeted Ad-mediated *BMPR2* transgene delivery to defective pulmonary endothelium may be a potential causal therapeutic approach to repair endothelial BMPR2 signalling. Feng et al. demonstrated that intravenous delivery of AdBMPR2-myc to transgenic mice harbouring a dominant negative *BMPR2* mutation (R889X), known to cause PAH, reversed right ventricular systolic pressure and right ventricular hypertrophy highlighting a therapeutic effect [4]. AdBMPR2-myc is a first-generation E1/E3-deleted HAd-5 expressing human *BMPR2* from a CMV promoter [187]. To improve transgene delivery to murine pulmonary endothelium, a bi-specific conjugate was formed between the antigen-binding fragment of the 1D6.14 antibody (binds to knob on AdBMPR2-myc) and the murine 4B10 antibody which recognises the angiotensin-converting enzyme which is highly expressed on pulmonary endothelial cells (ECs) [188]. In addition, systemic HAd-5 delivery may be circumvented by tracheal administration of nebulised VPs [189]. Pozeg et al. showed that tracheal delivery of nebulised replication-deficient HAd-5 Kv1.5 (encodes Kv1.5 potassium channel, an O_2_-sensitive channel which is downregulated in chronic pulmonary hypoxia) normalised pulmonary vascular constriction/resistance in a rat model of PAH [189]. Watanabe et al. demonstrated that nebulised delivery of AAV-1 SERCA2A via the trachea ameliorated pulmonary vascular remodelling and resistance in a porcine model of PAH [190]. Together, these findings may warrant a study investigating tracheal administration of nebulised recombinant HAd-5 BMPR2 in a porcine model of PAH.

## Secondary lymphoedema

Approximately, 20% of breast cancer patients who undergo axillary lymph node dissection and radiotherapy develop secondary lymphoedema [191]. The mechanical interruption of upper limb lymph drainage leads to accumulation of interstitial fluid and pitting oedema resulting in irreversible accumulation of fibro-adipose tissue and non-pitting oedema [192]. Although surgical vascularised lymph node transfer (VLNT) is effective in reducing secondary lymphoedema [193], implanted lymph nodes incorporate at low frequency into existing lymphatic vasculature, requiring patients to continue wearing compression garments [194]. A phase I trial sponsored by Herantis Pharma Plc. (Espoo and Turku, Finland) aimed to investigate the safety and tolerability of perinodal AdAptVEGF-C ex vivo injection during VLNT in 15 breast cancer patients [192]. AdAptVEGF-C (Lymfactin®) is a first-generation replication-deficient E1/E3-deleted HAd-5 which contains the CMV promoter driving human *VEGFC* transgene expression [195] shown to induce lymphangiogenesis in lymph node-excised mice [196]. After a 12-month follow-up, the phase I trial demonstrated that Lymfactin® appeared to be well tolerated [192]. Importantly, the investigators did not detect viral DNA in patients’ bloods or an increase in Lymfactin®-specific antibodies following perinodal ex vivo injection. This phase I trial is continuing with a planned 3-year efficacy and a 5-year safety follow-up. Since this study is not placebo-controlled, it cannot determine whether perinodal ex vivo Lymfactin® injection during VLNT has a beneficial effect compared to VLNT alone. Hence, Herantis Pharma Plc. has initiated a larger randomised placebo-controlled phase II trial (AdeLE) which aims to determine Lymfactin® efficacy in 39 breast cancer patients.

## Barriers to successful adenoviral cardiovascular gene therapy in humans and potential solutions

Despite considerable advances in cardiovascular gene therapy research, many barriers remain in place. Potential strategies of overcoming these hurdles will be discussed in the following section.

## Evading host immune response and targeted delivery

Immune-mediated vector clearance and targeted transgene delivery to cardiovascular tissue remain significant obstacles for cardiovascular gene therapy. Local delivery routes for HAd-5 vectors such as intracoronary injection and direct myocardial injection as well as ex vivo perinodal injection or intralumenal transduction of SVGs circumvent the need for systemic vector delivery which reduces the risk of potentially deleterious side effects. Specifically cardiac gene therapy trials in humans relying on local vector delivery have, if at all, only ever shown modest therapeutic effects. One reason for this may be explained by immune-mediated clearance of therapeutic vectors before they arrive at their respective targets. Given the high prevalence of HAd-5 and AAV nAbs in the population, it would be beneficial to integrate nAb screening as a potential exclusion criteria into all future cardiovascular gene therapy trials. Importantly, it may be speculated that introduction of immune modulators such as tacrolimus [93], cyclophosphamide [92], or imlifidase [94] to future cardiovascular gene therapy trial designs may enhance transgene delivery. This may prove to be particularly useful in settings where repeated local HAd-5 gene therapy vector delivery may be necessary to maintain a treatment effect given the limitation of transient transgene expression. In addition, the use of rare human or even xenogenic Ads (low levels or absence of nAbs) as well as Ad capsid/fibre modifications (pseudotyping) potentially in combination with immunomodulation may avoid immune-driven clearance and enable more targeted transgene delivery to cardiovascular tissues and should, therefore, be considered for future trials in humans. It is noteworthy to mention that the manufacturing process for these alternative Ad serotypes has matured and that authorised adenoviral-based COVID-19 vaccines have provided the evidence that alternative Ad serotypes (including non-human) are clinically applicable in humans (see Table 4).

**Table 4:** List of current Ad vector-based vaccine development programmes

Start and finish date	Indication	Product name	Viral vector, dose regimen, administration route	Number of patients	Phase	Clinical trial number	Efficacy	Serious adverse events reported in treatment arm	Company/university
09/2020 until 03/2023	SARS-CoV-2		Ad26.COV2.S, 1 dose, IM	*N* = 43,783	III	NCT04505722 [22]	63.7 to 76.7% (≥ 14 days after administration) 62.0 to 85.4% (≥ 28 days after administration)^1^	DVT (*N* = 6), PE (*N* = 4), TST (*N* = 1), seizure (*N* = 4), tinnitus (*N* = 6) [22] Thrombotic thrombocytopenia [199]	Janssen (Belgium)
05/2020 until 08/2021	SARS-CoV-2		ChAdOx1, 2 doses, IM	*N* = 23,843	II/III	NCT04324606 NCT04400838 NCT04444674 [24]	70.4% (> 14 d after 2n^d^ vaccine dose)	Transverse myelitis (*N* = 1, independent neurological committee considered idiopathic, short segment, spinal cord demyelination as most likely diagnosis), 40 °C fever (*N* = 1)[24] Thrombotic thrombocytopenia (*N* = 11) [200]	Jenner Institute (Oxford, UK), AstraZeneca (UK)
03/2020 until 02/2021	SARS-CoV-2		Ad5 vectored COVID-19 vaccine, 1 dose, IM	*N* = 108	I	NCT04313127 [66]	Study did not investigate efficacy. Vaccine triggered rapid T-cell and humoral responses against SARS-CoV-2	Fever > 38.5 °C (*N* = 9)	CanSino Biologics Inc. (China)
09/2020 until 05/2021	SARS-CoV-2	Gam-COVID-Vac	rAd26.S, 1st dose rAd5.S, 2nd dose Both IM	*N* = 21,977	III	NCT04530396 [20]	91.6%	No vaccine-related SAEs reported	Gamaleya Research Institute of Epidemiology and Microbiology, Health Ministry of the Russian Federation (Russia)
12/2019 until 01/2021	MERS-CoV		ChAdOx1 MERS, 1 dose, IM	*N* = 24	Ib	NCT04170829 [201]	Study did not investigate efficacy. Vaccine triggered T-cell and humoral responses against MERS-CoV	No SAEs reported	King Abdulaziz Medical City, National Guard Health Affairs (Riyadh, Saudi Arabia)
08/2019 until 12/2021	Ebola	Ad26.ZEBOV/MVA-BN®-Filo	Ad26, 2 doses, IM	*N* = 800	II	NCT04028349	Ongoing trial	Ongoing trial	Janssen, CEPI
10/2019 until 03/2024	HIV	Ad26.Mos4.HIV	Ad26, 4 doses, IM	*N* = 3902	III	NCT03964415	Ongoing trial	Ongoing trial	Janssen
09/2020 until 10/2021	SARS-CoV-2	VXA-CoV2-1	Ad5, 1 dose, oral	35	I	NCT04563702	No publication yet	No publication yet	Vaxart (USA)

*IM* intramuscular, *DVT* deep vein thrombosis, *PE* pulmonary embolism, *TST* transverse sinus thrombosis, *SAE* serious adverse event, *CEPI* the Coalition for Epidemic Preparedness Innovations

^1^Range depending on variable subgroup analysis

## Stable transgene expression

Stable transgene expression may be desirable in non-angiogenic cardiac gene therapy. Although, in theory, the use of LV vectors enables stable long-term transgene expression, the heightened risk of mutagenesis and the fact that no gene therapy to date is 100% cardiac-specific disqualify the use of LV vectors in human cardiac gene therapy trials at present. Based on stable long-term transgene expression and low mutagenesis risk, AAV-based vectors theoretically appear to be good candidates for cardiac gene therapy aiming for stable long-term transgene expression. However, the CUPID-2 trial did not detect a significant therapeutic benefit of AAV-1/SERCA2A for patients with HFrEF. The authors detected low vector DNA levels in a small number of cardiac samples suggesting that transgene delivery was inefficient following intracoronary vector administration (1 × 10^13^ VPs). Furthermore, the absence of AAV-1 nAbs and mostly negative CD8 + T-cell responses made immune-driven vector elimination unlikely. One may argue that a higher VP dose may be viable and could potentially improve transgene delivery. Contrasting these findings, Kawase et al. demonstrated that intracoronary administration of AAV-1/SERCA2A at the lower dose of 1 × 10^12^ VPs was enough to reverse volume overload HF and induce sustained cardiac *SERCA2A* expression in pigs, admittedly at 2-month follow-up [155]. As mentioned in the previous section, repeated intracoronary administration of HAd-5 or alternative Ad-based vectors ± , the potential use of immunomodulators to overcome the limitation of transient transgene expression may provide a more effective strategy to achieve a stable long-term cardiac transgene expression.

With regard to detecting cardiac transgene expression following vector administration in humans, it must be pointed out that the inability to routinely determine transgene expression in cardiac tissue remains a major limitation. Investigators of previous cardiac gene therapy trials were only ever able to determine transgene expression in a limited number of samples obtained from either deceased patients or patients having to undergo surgical procedures. The significant risk associated with cardiac biopsies, especially in HF patients, has led to the view that this method cannot be routinely used to ascertain cardiac tissue samples from trial participants. Given the absence of safe routine sampling, there remains a large unknown for the foreseeable future. With regard to angiogenetic cardiac gene therapy, one potential way of addressing this issue would be to introduce myocardial perfusion imaging (MPI) by positron emission tomography (PET) as a surrogate marker of angiogenesis to future trials. PET is considered the gold standard of MPI (reviewed in) [197]. Compared to SPECT used in the AGENT4 trial, PET provides superior image quality, higher spatial resolution enabling detection of smaller/subtle perfusion defects, higher temporal resolution allowing for absolute quantification of perfusion, lower radiation burden, and acquiring rest and stress images within a single scanning session.

## Pro-oncogenic safety concerns in angiogenic cardiac trials

Delivery of first-generation replication-deficient HAd-5 vectors carrying therapeutic transgenes via intracoronary infusion and minimally invasive transthoracic/catheter-based intramyocardial injection has been shown to be at least safe in the short term in humans. Angiogenic cardiac gene therapy trials to date have relied on HAd-5-mediated delivery of the pro-angiogenic proteins VEGF, FGF4, and HGF which all also display pro-oncogenic characteristics. Hence, there remains a real concern around potentially promoting a pro-oncogenic environment following transgene delivery. Encouragingly, the development of AdVEGF-All6A + (XC001) which drives expression of the less pro-oncogenic VEGF isoform 189 may reduce the potential risk of creating a pro-oncogenic environment. A trial sponsored by Weill Medical College of Cornell University will help to determine long-term safety (10 years)/efficacy of XC001.

## Study design and disease burden

Major limitations of most previous cardiovascular gene therapy trials included low numbers of trial participants, short-term follow-up, and advanced disease burden. In combination with likely inefficient gene transfer, these conditions make it very difficult to detect a significant treatment effect which may only be small. This is especially true in advanced HF with severe disease burden in often co-morbid study participants which may partially explain the discrepancy between pre-clinical and clinical findings using the same gene therapy vector at comparable doses. This bias may be addressed two-fold. On the one hand, it would be beneficial to be able to include patients with less HF burden and less co-morbidities in future trials. On the other hand, pre-clinical animal models may have to change to more accurately reflect human disease. In addition, larger cohorts (similar to CUPID-2, *N* = 250) will facilitate subgroup analysis and potentially elicit small/subtle parametric changes. The AFFIRM study aims to recruit 160 participants to determine the safety/efficacy of Generx® in a larger population.

## Summary

Gene transfer vectors based on human adenovirus serotype 5 were some of the first developed viral gene therapy products which were translated into human clinical trials in a range of cardiovascular diseases. As knowledge about adenoviruses increased, other alternative serotypes have been identified in experimental research, some of which have been translated into clinical use. Robust and optimised manufacturing processes which meet all regulatory release requirements are well established for GMP adenoviral vector production, and the recent COVID-19 pandemic has demonstrated the highly significant potential that gene transfer vectors based on adenoviruses have for use as mass medicines in the human population.
